# Embedding the public voice in clinical trials: developing public-informed training for patient data research

**DOI:** 10.1186/s40900-026-00928-y

**Published:** 2026-07-06

**Authors:** Fiona V Lugg-Widger, Caroline Brocklehurst, Sarah Chave, Linda Farey, Araya Gautam, Edward Goodall, Michelle Abhayawardena Goonasekera, Daniel Iheanacho, Rashmi Kumar, Joanne Lloyd, Isla Mackenzie, Marion Mafham, Clara Martins de Barros, Elizabeth McCall, Stephen T Moore, Kim Munnery, Macey Murray, Nicholas Pitt, Yasmin Rahman, Sarah Rawlinson, Michael Robling, Amy Rogers, Kamil Sterniczuk, Robert Trubey

**Affiliations:** 1 Centre for Trials Research, Cardiff University, Cardiff, UK; 2 HDR UK Transforming Data for Trials Public Advisory Group, London, UK; 3 Oxford Population Health, University of Oxford, Oxford, UK; 4 MEMO Research, School of Medicine, University of Dundee, Dundee, UK; 5 MRC Clinical Trials Unit at UCL, Institute of Clinical Trials and Methodology, University College London, London, UK; 6 DECIPHer, Cardiff University, Cardiff, UK

**Keywords:** Patient and public involvement, Clinical trials, Capacity building, Health systems data, Co-production, Participatory health research, Impact of public involvement

## Abstract

**Background:**

The use of health systems data (HSD) such as patient records and prescriptions is expanding within clinical trials to improve efficiency, reduce participant burden, and enhance real‑world relevance. However, challenges remain around governance, data quality, transparency and public trust, echoed through discussions in this project. While Patient and Public Involvement and Engagement is increasingly embedded in trial design, involvement in methodological and data‑focused areas remains limited. Ensuring that HSD trial training for researchers and public reflects public perspectives is therefore important.

**Methods:**

This work was conducted within HDR UK’s Transforming Data for Trials programme. A Participatory Health Research (PHR) approach was used to develop training resources for the HDR UK Futures online learning platform. A UK‑wide Public Advisory Group (PAG) was recruited through national involvement networks. Engagement took place through virtual full-PAG and small-working‑group meetings. PAG members were involved in identifying priorities, shaping content, reviewing materials and recording videos. Two case studies illustrate different approaches to involvement: co‑production throughout development of a public‑facing module and consultative input to a researcher‑focused module.

**Results:**

The PAG comprised 25 members with diverse geographical and experiential backgrounds. Case Study 1: members helped develop eight short videos for public partners. They influenced topic selection, tone, language and accessibility of scripts; six members also participated in filming. Their input led to clearer explanations of topics including data governance, consent and trust, with content presented in plain English and modular formats. Case Study 2: PAG members provided feedback on a technical training module on Data Utility Comparison Studies which compare the usefulness of different data sources. Their feedback highlighted concerns about data accuracy, transparency and potential equity implications, prompting refinements to the framing and examples used within the module.

**Discussion:**

Co‑production supported the development of accessible resources for public research partners, while consultative input helped ensure researcher‑focused training addressed issues relevant to public trust and accountability. These approaches demonstrate how public perspectives can strengthen training related to HSD trial methodology.

**Conclusion:**

Embedding public perspectives in HSD training development could enhance relevance, accessibility and trust. This work provides a practical model for involving public contributors in methodological training within trials.

**Supplementary Information:**

The online version contains supplementary material available at 10.1186/s40900-026-00928-y.

## Abstract (plain language summary)

Clinical trials are studies that test new treatments or ways of caring for people. Increasingly, these trials use patient data, such as information from appointments, scans, referrals and prescriptions. Using these data can make studies faster and easier for people taking part and for researchers.

However, some still have questions about how:

• The data are used.
• Safe and accurate the data are.
• The data should be handled.

To help address these questions, our HDR UK trials team developed training materials about the use of patient data in clinical trials. These materials were created using a co-production approach, which means researchers and members of the public work together as partners. Members of a Public Advisory Group (PAG) contributed their experiences as patients, carers and public contributors to research throughout the development process. The PAG helped decide:

• What topics to include.
• How to explain the information clearly.
• How to present it in an easy-to-understand way.

We give two examples of their work. First, the PAG co-produced a set of short videos for new public contributors. Second, they advised on a technical training module for researchers. The training is freely available on the HDR UK Futures platform (registration required): https://hdruklearn.org/.

Working with the PAG made the training more relevant, easier to follow, and based on what the public cares about. Their input also highlighted important issues like the need for trust, data security, accuracy, and fairness. This example shows that involving the public, even in technical areas, can improve the outputs for everyone.

**Supplementary Information:**

The online version contains supplementary material available at 10.1186/s40900-026-00928-y.

## Background

### Health systems data in clinical trials

Clinical trials increasingly rely on health systems data (HSD), such as patient records, scans, referral letters and prescriptions, to make research more efficient, reduce participant burden, and generate evidence that better reflects real-world care [1]. These data offer substantial opportunities, but their use in trials remains complex and often poorly understood outside specialist communities.

Despite strong interest in using HSD in trials, researchers continue to face challenges around data quality, access, governance, transparency and public trust [2–4]. These issues can undermine the acceptability of HSD-enabled trial designs among researchers, regulators, clinicians, and the public. Training that reflects the needs of all these groups and is available to them is therefore essential to support responsible and confident use of HSD.

### Patient and public involvement in methodological research

While Patient and Public Involvement and Engagement (PPIE) is now well established across many aspects of clinical trial design and delivery, opportunities for public contributors to influence methodological and data science components have historically been more limited. Meaningful public involvement happens when members of the public (patients, carers, service users, or community members) work with researchers as partners, shaping decisions, influencing design, and improving how research is conducted and shared.

Evidence indicates that researchers often cite the technical nature of these domains, alongside uncertainty about the relevance of PPIE and a lack of practical guidance on how to involve public contributors effectively, as key barriers to involvement [5, 6]. Previously published work suggested that PPIE within methodological, statistical, and data-intensive research is frequently overlooked or undervalued, leading to limited representation of public perspectives in decisions that shape trial design, analysis, and data use [7]. This underrepresentation highlights a critical gap: the development of training for health data science within trials must actively involve public contributors to ensure that resulting resources are not only technically robust but also socially informed, ethically grounded, and aligned with public values.

### Developing public-informed training for trials using health systems data

To support the research community in addressing these challenges, this work was conducted within the HDR UK *Transforming Data for Trials* programme (2023–2029) [8]. The programme aims to build UK-wide capacity for the use of health systems data in clinical trials through training, tools, and infrastructure. The courses and training work package focus on developing training resources for both public contributors and researchers that reflect the practical realities of working with HSD. These resources are hosted on the HDR UK Futures platform [9], which provides freely accessible, registration-based, video-led modular courses aligned to key stages of the clinical trial *‘Routemap’* [10].

A Public Advisory Group (PAG) was established to provide participatory input across the programme, including training development. The PAG’s objectives were to support the identification of training priorities, shape content and delivery, review materials, and help promote training outputs to public contributors, trial participants and wider public audiences. This paper describes how these objectives were implemented through the development of training resources. We outline our partnership with the PAG, describe the participatory methods used to co-create training content, and present two case studies illustrating contrasting approaches to embedding public voice, ranging from targeted consultation to full co-production.

### Methods

Our work adopts a Participatory Health Research (PHR) approach to guide the development of training resources [11–13]. PHR has been defined as research conducted with rather than on people and seeks to maximise the meaningful involvement of those affected by or interested in the topic under study. It emphasises equitable stakeholder involvement throughout all stages of research and supports shared decision-making, mutual learning and co-production [14]. Applying PHR principles meant that public contributors were treated as partners rather than only as consultees, influencing module prioritisation, content, presentation, framing and in some cases, delivery.

Within PHR frameworks, participation operates on a continuum, from being informed, to consultation, collaboration and empowerment [15]. Decisions about the level of participation were initially made by the research team at each stage of development, ensuring that contributors’ time, expertise and preferences aligned with the purpose and needs of each module. PAG members were also able to influence how they were involved, and plans were adjusted in response to their feedback. This shaped the extent and nature of PAG influence across the two case studies presented.

### Public advisory group (PAG) recruitment and composition

An open call for expressions of interest was advertised through:

- HDR UK Voices – a UK-wide community of public contributors interested in health data [16];
- NIHR People in Research – the national registry for public involvement opportunities [17];
- Health & Care Research Wales public involvement network [18];
- Administrative Data Research (ADR) UK – a national partnership supporting safe and ethical use of administrative data [19].

These networks were selected for their extensive national reach and relevance to data-driven research.

A total of 115 individuals from across the four UK nations responded, with 25 invited to become PAG members. Selection prioritised diversity in age, ethnicity, gender, educational background, lived experience (e.g. as patients or carers) and experience with clinical trials or HSD.

### The public advisory group (PAG) partnership

Structured engagement was achieved through online full PAG meetings and smaller working group meetings. Full PAG meetings (twice-yearly) provided programme updates and discussion of overarching priorities, while ad-hoc smaller working group meetings (typically 6–8 members) focused on specific work-packages or topics. Online delivery enhanced accessibility and enabled contributors to participate flexibly alongside other commitments. Materials were circulated in advance and meetings followed clear agendas to support preparation and participation.

Effective public involvement required dedicated investment. PAG members were offered remuneration for their time and contribution in line with NIHR guidance, and staff resources were allocated to coordinate meetings and support contributors. The approach to involvement also evolved in response to PAG feedback, with adjustments made to communication, meeting formats and opportunities for participation. These partnership structures will continue until the programme concludes in 2029.

### Case studies

This paper presents two case studies (Table 1) that illustrate contrasting approaches to working with the PAG in the development of training modules. The first case study describes co-production throughout the development of a public-facing video module. In contrast, the second case study describes a consultation-based approach for a researcher-focused module. Together, these case studies show that different levels and forms of public involvement may be appropriate depending on the aims of the training, the intended audience and the nature of the content being developed.

**Table 1:** Training modules: overview and target audiences

	Case study 1	Case study 2
Module title:	Trials using Patient Data: A Resource Hub for Public Research Partners	Data utility considerations for clinical trials
Link	https://hdruklearn.org/courses/course-v1:HDRUK+RP001+2026	https://apps.hdruklearn.org/learning/course/course-v1:HDRUK+DUCCT001+2025/home
Audience:	Members of the public interested in being involved as a lay member / public contributor for trials using HSD	Trialists using HSD
Overview of the training:	This resource comprises a series of standalone topics covering key activities in clinical trials that use HSD. The content focuses on areas where public contributors most commonly support trials and aims to build confidence while providing an overview of tasks that may arise across the trial lifecycle. The module includes eight short videos: 1. Understanding clinical trials: how patient data can make a difference 2. Becoming a public contributor: your role in clinical trials using patient data 3. How patient data are kept safe in clinical trials 4. Understanding consent and participant information in trials using patient data 5. Applying to access patient data for trials: a guide for public contributors 6. How trial outcomes are measured using patient data: a guide for research partners 7. From trial to archive: understanding patient data after a trial 8. Data governance in clinical trials: a public contributor’s guide	This course provides an introduction to Data Utility Comparison Studies (DUCkS), focusing on their purpose, benefits, challenges, and practical applications in clinical trials. It explores methods for comparing HSD with trial-collected data, including retrospective and prospective approaches, and drawing on real-world case studies. The module includes six videos: • An Introduction to Data Utility Comparison Studies • What is a Data Utility Comparison Study? • Benefits and Challenges of Data Utility Comparison Studies • Planning a Data Utility Comparison Study • Data Analysis and Reporting for Data Utility Comparison Studies • An Overview of Three Data Utility Comparison Studies

### Case study 1: Co-produced public-facing training module

The first case study focuses on a training module comprising a series of short videos aimed at public research partners. The initial idea for the module was proposed by the research team and brought to the first full PAG meeting as a discussion point. During the meeting, several potential training topics and audiences were discussed, and PAG members identified a resource to support public contributors involved in trials using health systems data as a key priority. PAG members identified the idea as a key priority and helped shape the direction of work. The module introduced key trial and data concepts in an accessible way and was subsequently refined through sustained public contributor involvement at every stage of development. Most PAG members contributed at different stages of development, bringing diverse perspectives to the work. Four 90-minute meetings focused on topic prioritisation, script development, review, and feedback, followed by three additional sessions dedicated to recording the final scripts.

### Case study 2: Public consultation informing research-focused training

The second case study, targeted at researchers, was developed by the research team. Public involvement was embedded through a consultative approach, with PAG members contributing to the written content to ensure it was grounded in public values and perspectives, particularly in relation to trust and equity. One 90-minute meeting was held with five members of the PAG.

## Results

### Characteristics of the public advisory group (PAG)

The 25-member PAG reflects a broad geographical distribution across all UK nations. Over half of members (54%) were based in England, with 18% each in Scotland and Wales, and 10% in Northern Ireland.

The PAG spans a broad age range, with most members aged 45–64 (49%), followed by 25–44 (22%), over 65 (13%), and under 25 (4%).

Approximately two-thirds of PAG members identify as women. Recruitment and selection aimed to achieve diversity in ethnicity and educational background, alongside age, gender and geography, to ensure a range of lived experiences and perspectives relevant to the programme.

### Case study 1: Co-production of a public-facing video module

Four smaller working group meetings were held over six-months with a total of 14 PAG members. Through focused discussion, members helped shape the topics covered, as well as the narrative, tone, and structure of the videos. Expressions of interest were invited for each stage of development, with members selected based on availability and previous contributions to maximise opportunities for a broad range of PAG members to be involved. Eight members reviewed and edited draft scripts, and six participated in video recordings to enhance authenticity and representation.

#### Availability and style of training resources

The early smaller working group meetings reflected on the training the PAG members had when working on other research studies as public contributors, how there was no standard set of training available, with some trials providing a suite of training and access to resources and others, none at all. There was recognition that training/induction is important as *“[you] can’t input into the patient information leaflet if you don’t understand the study yourself”.*

The style and delivery of the training were also considered highly important. PAG members emphasised the value of brief, bite-sized information that would build learners’ confidence rather than overwhelm them with too much information. It was noted that learners may be unwell or need additional support (such as sensory needs, neurodivergence or language considerations) so the training materials needed to be tailored to them. While training was recognised as an important part of being involved in a research team, it was also acknowledged that training should not be delivered in isolation. The trial team remains responsible for ensuring that public contributors are well supported, including maintaining regular contact and providing ongoing opportunities for support and connection with both the trial team and other public contributors.

#### Agreeing the eight videos

Key themes of topics for training emerging from discussions included:

- Explaining a public contributor’s role and expectations upfront
- Addressing concerns about data security, consent, and governance
- Describing the process of how the data is collected, accessed and used

These insights informed the development of eight short videos; each designed as a stand-alone resource (Table 1). Draft scripts were written by the research team and pre-circulated to a smaller working group of PAG members. All comments and edits were considered ahead of the meeting and then discussed for clarity, ensuring the changes addressed the suggestions being made. Common feedback included the need for definitions and accessibility of the language used, including adapting the readability of some sections.

Table 2 demonstrates the participatory process by showing how contributors’ feedback directly shaped the training content. The iterative feedback loop ensured that the final resources were accessible, relevant, and grounded in public values.

**Table 2:** Mapping feedback themes to video content

Feedback Theme	How It Was Incorporated	Video(s)* Impacted
Need for clear definitions and accessible language	Added glossary terms and simplified technical language in scripts; ensured plain English readability	All eight videos
Clarify contributor roles and expectations	Developed introductory videos explaining the tasks and responsibilities of public contributors	Videos 1 & 2
Explain data security and governance	Created dedicated content on secure data environments, pseudonymisation, and governance processes	Videos 3 & 8
Address negative media coverage and trust issues	Framed messaging around transparency and safeguards	Videos 3, 6 & 8
Provide clarity on consent and data use	Included explanations of consent processes and what data is collected and why	Videos 4 & 5
Explain post-trial data handling	Added content on archiving and long-term data storage	Videos 7
Highlight equity and accessibility considerations	Ensured tone and delivery were inclusive; videos were designed as short, standalone modules to support flexible viewing. All videos included subtitles and minimal background music.	All eight videos

*See Table 1 for details of each video

#### Development and filming of training content

PAG members were offered the option for their sessions to be recorded using either audio-only or video formats. This choice was provided to support contributor comfort and autonomy. In collaboration with the HDR UK Training Team, contributors received preparatory guidance in advance of the sessions, including practical advice on lighting, clothing, and camera positioning. Participant-facing materials, including a crib sheet and short scripted clips (approximately three minutes of content), were shared ahead of recording.

Three recording sessions were held, with six public contributors taking part across the sessions. Facilitator packs were developed to support the structured delivery and monitoring of the sessions and contributors received tester training beforehand to allow for questions and clarification. During the sessions, scripts and materials were adapted in real time (e.g. using larger fonts, adding explainers, and offering tips on positioning documents on screen to improve readability and eye line) supporting an accessible and collaborative filming environment. All contributors provided informed consent and signed a release form and reported positive experiences of participation.

### Case study 2: Embedding the public voice in researcher-focused training

The second training module, titled Data Utility Considerations for Clinical Trials, is aimed at trialists working with HSD [20]. This course introduces the concept of Data Utility Comparison Studies (DUCkS) [21] which evaluate the suitability of HSD for trial outcomes by comparing it with data collected directly within clinical trials. The module covers their purpose, benefits, challenges and practical applications, using real-world examples to demonstrate how DUCkS can be designed, conducted and interpreted in practice.

While the module scope was predefined, public involvement influenced its framing and emphasis. Public involvement was integrated through a dedicated meeting with four PAG members, where feedback was solicited on the module’s content/emphasis and framing. Although the topic had already been identified, the group’s input was instrumental in shaping the final version of the training ensuring that the module addressed public perspectives on data quality, equity and trust alongside methodological considerations.

#### PAG perspectives on data utility comparisons

Discussion with PAG members highlighted three key areas of reflection on the use of HSD for trial outcomes.

First, members raised concerns about the accuracy of medical records, informed in part by their personal experiences of accessing primary care records via the NHS app. Members expressed apprehension that trials relying on HSD for outcomes might be biased if the underlying data were incomplete or inaccurate. This was based on their own experiences of receiving inaccurate letters or records of missed appointments that were incorrect. Members also reflected on the broader importance of ensuring that data are sufficiently comprehensive and reliable to support public trust in trial findings.

*I’m really keen in general that researchers make sure that any data is broad enough*,* comprehensive enough and reliable enough to create quality outcomes. Gaining and maintaining public trust and how the data might or might not be reliable… I think those things are really intrinsically intertwined actually*,* both rely on each other. ***PAG member 4**,** data utility comparison meeting**,** June 2024**

Second, PAG members emphasised equity considerations. They highlighted the potential for existing biases in medical reporting and diagnoses to be reflected within health records, particularly for conditions that are known to be under-recognised or inconsistently recorded. Discussions also noted the potential exclusion of groups who do not routinely engage with health services.

*I think that women face a lot of gender bias in especially in medical diagnosis and treatment. So if we think about things like symptoms for conditions like heart disease*,* which are under-recognised or often attributed to something else*,* like usually psychological causes*,* yeah. So I expect that to lead to incomplete or inaccurate records. And also the underreporting… [of] things like endometriosis and polycystic ovary syndromes. ***PAG member 2**,** data utility comparison meeting**,** June 2024**

Alongside these concerns, PAG members recognised the potential value of data utility comparisons. Comparing trial-collected data with routinely collected health data was viewed as a way to assess data quality, improve transparency, and support public trust in the use of health data within trials.

*We’re talking about the negatives*,* but I think there are positives… these DUCkS could show people that we are trying to compare the trial gathered data and the NHS data (the secondary data) to try and increase the reliability. So I think it’s important to explain to people how these DUCkS can improve our confidence and give examples of when it could be helpful. ***PAG member 3**,** data utility comparison meeting**,** June 2024**

These insights were directly incorporated into the training materials. The final video scripts framed DUCkS not only as a technical exercise but also as a response to public concerns about data accuracy and trust. The training emphasised that comparing HSD with trial-collected data is a critical step in ensuring transparency and building public confidence in research practices. Additionally, the module addressed equity implications, acknowledging that reliance on HSD may inadvertently reinforce existing disparities in healthcare access and documentation.

### Reflections from the public advisory group (PAG) co-authors

As co-authors and members of the PAG, we reflected on our experience of contributing to the development of training on the use of patient data in clinical trials. Our key message is patient and public voices should be actively considered and are essential for any effective outcome. When public members are provided with a respectful and supportive environment and training, they can make valuable contributions to topics that may initially appear highly technical. Many of us began with limited familiarity with health data governance or trial methodology, but clear explanations and open discussion created a safe space to ask questions and share perspectives. PAG members brought not only their own experience, skills and professional backgrounds but also the experience of wider communities and networks, ensuring the diversity of public contributors can strengthen the effective impact of the research. Importantly, while the initial concept for the training videos did not originate from the PAG, members were involved from a very early stage and played a central role in shaping and developing the idea, highlighting the value of public contributors not only in refining outputs but also in influencing their direction from the outset. When properly supported and trained, and given opportunities to learn and build confidence, public contributors can make valuable contributions to research. Such learning opportunities may also support sustained engagement in future projects.

PAG involvement directly influenced the training materials, shaping the length, language, tone and style of the videos, resulting in shorter, accessible “bite-sized” resources delivered through a peer-to-peer approach. Without public input, the materials risked being overly technical or out of touch with the intended audience, potentially reducing their effective use and impact.

Our discussions also highlighted questions the wider public have about the use of patient data, including what role individuals have and how permission (or consent) are considered in trials relying on patient data, and how privacy and anonymity are protected. Concerns about commercial use of data and expectations for transparency, particularly among younger generations, were also identified and considered. Working together in this way shows how investing in meaningful, properly resourced, accessible and flexible patient and public involvement can strengthen research, bringing real-world perspective and “*life*” to outputs while helping contributors better understand how health data research works, and generating learning that can be shared and adapted across other projects.

## Discussion

This paper demonstrates how principles of PHR can be operationalised in the development of training resources for clinical trials using HSD. Core PHR principles, including shared decision-making, co-learning, reflexivity, and attention to power dynamics, were embedded through flexible engagement structures, iterative feedback and the active involvement of PAG members in shaping both content and delivery [15]. Across both case studies, these principles were applied in ways that were responsive to the intended audience and purpose of each training resource, illustrating how participatory approaches can be adapted rather than uniformly applied.

Together, the two case studies illustrate how public involvement strategies may be tailored to different contexts. Importantly, impact was evident across both case studies despite differing levels of participation, demonstrating that consultation and co-production can each contribute valuable insights when aligned with the aims and context of the training. The co-produced public-facing module represents a model of deep participation, aiming to produce resources that are accessible, trustworthy, and aligned with contributors’ priorities. In contrast, the researcher-focused module demonstrates how more limited, consultative involvement can still shape technical content, particularly by encouraging greater attention to issues of public trust, transparency and equity within methodological discussions. These examples reflect wider participatory health research literature, which recognises that public involvement operates along a continuum, from consultation to collaboration and empowerment.

### Strengths and limitations

A key strength of this work was the flexible and responsive approach to public involvement. This paper is reported in line with GRIPP2 guidance, ensuring transparent and comprehensive reporting of patient and public involvement throughout the manuscript. The use of structured meetings, opt-in participation, and subgroup formats enabled contributors to engage in ways that reflected their interests, availability and confidence. These participatory approaches fostered a sense of shared ownership and collaboration, which in turn enhanced the credibility and relevance of the resulting training materials. A further strength was the diversity of the PAG, which brought together individuals with a range of lived experiences, health conditions, levels of research experience, and backgrounds from across the UK. This diversity enabled a broad range of perspectives to inform the development of the training materials and helped ensure that the outputs were relevant and responsive to the needs of different audiences. Participation was also designed to be accessible and inclusive. Members were able to join meetings remotely, participate via chat or discussion, and choose whether to use cameras or microphones. Materials were shared in advance, and meetings followed clear agendas and timings with appropriate breaks, supporting preparation and enabling members to participate in ways that suited their needs and preferences.

Nonetheless, the timing and scope of public involvement in the researcher-focused module could be strengthened by involving contributors earlier and at additional stages of the development process, such as co-shaping the learning objectives. While both training topics originated within the research team as part of the wider programme of work, future initiatives could consider involving public contributors earlier in identifying training needs and priorities where feasible. Future work should also explore how these approaches can be adapted for use by individual trial teams operating outside established PPIE infrastructures. While the PAG included diversity across several characteristics, further efforts are needed to ensure representation from underserved and marginalised communities. This is particularly important when addressing the use of health systems data, where structural inequalities in data representation and access may otherwise remain insufficiently recognised or challenged.

### Impact on the development of the training resources

At the time of writing, evidence of impact varies across the two case studies due to differences in launch timelines and opportunities for evaluation.

The public-facing module (Case Study 1) was launched in early 2026 on the *HDR UK Futures* platform, and dissemination activities are currently underway. Through an iterative co-production process, PAG members helped shape both the content and presentation of the module. Their input aimed to ensure that the training addressed genuine informational needs and was pitched at an appropriate and accessible level for public research partners. Evaluation of the module’s reach and impact is reviewed on an ongoing basis using the course metrics (users, average watch time, survey responses) captured via Futures.

For the researcher-focused module (Case Study 2), launched in April 2025, public input informed both the content of the training and its subsequent dissemination. Feedback from PAG members was incorporated into academic presentations and training events, including a workshop at the International Clinical Trials Methodology Conference in 2024 and a series of webinars hosted by University College London and the MRC–NIHR Trials Methodology Research Partnership. With consent, video clips and quotations from PAG members were used to amplify public perspectives, helping to humanise methodological discussions and foreground trust as a central consideration in the use of health systems data. Audience feedback suggested that the inclusion of public voices reframed DUCkS not merely as a technical exercise, but as a practice with an important trust-building function.

Although full co-production was not feasible for Case Study 2, PAG members nonetheless played a meaningful role in shaping the framing, emphasis, and language of the module. This illustrates how public perspectives can contribute to highly technical, researcher-focused training and how different modes of involvement can generate meaningful impact when aligned with the aims and constraints of the training context.

### Evaluation and future directions

Formal evaluation of the training modules is an important next step. Planned activities include feedback surveys, usage analytics, and follow-up interviews with both contributors and trainees. As the modules were developed within the HDR UK Transforming Data for Trials programme and hosted on the HDR UK Futures platform, evaluation could also explore how perceptions of credibility, trustworthiness and relevance are influenced by the training provider, delivery platform and local context of use through targeted survey questions or brief interviews with trainees. Sustaining participatory approaches beyond initial development, through ongoing feedback mechanisms and opportunities for capacity building, will be important to maintain the inclusivity and relevance of the training over time.

This work also highlights several mechanisms through which participatory approaches may enhance trust in trials using HSD. Trust may be strengthened directly, by making visible that training resources are co-designed with public contributors; semi-directly through the inclusion of public voices within training materials (e.g. videos and quotations); and indirectly, by ensuring that content responds to public concerns and priorities. For new public research partners and trial staff, training that visibly embeds public perspectives may reinforce confidence that data practices are transparent, accountable, and socially grounded. PAG involvement across the programme continues to strengthen the public voice, shaping outputs beyond training, including the stakeholder prioritisation forum Green Paper [4].

## Conclusion

This work demonstrates that embedding public perspectives through participatory health research principles could enhance the development of training resources for clinical trials using HSD. By involving public contributors as partners rather than consultees, the resulting materials were not only technically robust but also designed to be accessible, trustworthy, and responsive to societal concerns. The approaches described here provide a practical model for incorporating public perspectives into methodological and data-intensive research, areas in which public involvement has historically been limited. Although developed for HSD-enabled trials, these approaches may help address barriers to public involvement across a range of data-intensive and methodological research settings. The resulting training resources have the potential to support public contributors in building confidence and understanding of complex topics, while helping researchers better recognise and incorporate public perspectives in the design and conduct of data-enabled research. As trials increasingly rely on health systems data, integrating public perspectives into how these methods are developed, explained, and taught will be essential to sustaining public trust and confidence in data-enabled research.

**Table:** 

Glossary of Terms	Definition
Clinical trial	A research study that tests treatments, interventions or ways of delivering care to determine whether they are safe and effective.
Co-production	An approach in which researchers and members of the public work together as partners throughout a project, sharing knowledge, experience and decision-making.
Data Utility Comparison Study (DUCkS)	A study that compares health systems data with data collected directly within a clinical trial to assess the suitability of routine data for trial outcomes and analyses.
Health Systems Data (HSD)	Data collected routinely through healthcare systems, such as electronic health records, hospital records, prescribing data, laboratory results and administrative datasets.
HDR UK Futures	HDR UK's online learning platform providing free training resources related to health data science and research.
Participatory Health Research (PHR)	An approach to research that actively involves people affected by or interested in the research topic as partners throughout the research process.
Public contributor	A member of the public who contributes to research through involvement activities, such as advising on study design, reviewing materials or helping disseminate findings.
Trial-collected data	Information collected directly from participants as part of a clinical trial, such as questionnaires, assessments or study-specific measurements.

## Supplementary Information

Below is the link to the electronic supplementary material.

Supplementary Material 1

## Data Availability

No datasets were generated or analysed during the current study.

## Abbreviations

ADR: Administrative data research
DUCkS: Data utility comparison studies
HDR UK: Health data research UK
HSD: Health systems data
MRC: Medical research council
NIHR: National institute for health and care research
PAG: Public advisory group
PHR: Participatory health research
PPIE: Patient and public involvement and engagement
